# Acute Administration of Clozapine and Risperidone Altered Dopamine Metabolism More in Rat Caudate than in Nucleus Accumbens: A Dose-Response Relationship

**DOI:** 10.3797/scipharm.0907-20

**Published:** 2009-11-29

**Authors:** Farhat Batool, Muhammad A. Haleem, Darakhshan J. Haleem

**Affiliations:** 1 Neurochemistry and Biochemical Neuropharmacology Research Laboratory, Dept of Biochemistry, University of Karachi, Karachi-75270, Pakistan; 2 Dept of Biomedical Engineering, Sir Syed University of Engineering and Technology, Karachi, Pakistan

**Keywords:** Atypical antipsychotics, Extrapyramidal side effects, Dopamine D2 receptors, Serotonin_1A_ receptors, Schizophrenia

## Abstract

The present study compares the extrapyramidal and neurochemical effects of clozapine and risperidone in rat caudate (corpus striatum) and nucleus accumbens (ventral striatum) dose-dependently. Animals injected with clozapine (2.5, 5.0 and 10.0 mg/kg IP) or risperidone (1.0, 2.5 and 5.0 mg/kg IP) *in acute* were sacrificed 1 h later to collect brain samples. Extrapyramidal side effects (EPS) in terms of locomotor activity and catalepsy were monitored in each animal after the drug or vehicle administration. Maximum cataleptic potentials were found only at high doses of clozapine (10.0 mg/kg; 60%) and risperidone (5.0 mg/kg; 100%). Neurochemical estimations were carried out by HPLC-EC. Both drugs at all doses significantly (p<0.01) increased the concentration of homovanillic acid (HVA), a metabolite of DA, in the caudate nucleus and decreased in nucleus accumbens. Levels of Dihydroxyphenylacetic acid (DOPAC) significantly (p<0.01) increased in the caudate by clozapine administration and decreased in the nucleus accumbens by the administration of both drugs in a dose-dependent manner. 5-Hydroxyindoleacetic acid (5-HIAA), the predominant metabolite of serotonin significantly decreased in the caudate and nucleus accumbens in a similar fashion. Levels of tryptophan (TRP) were remained insignificant in caudate and nucleus accumbens by the injections of two drugs. In caudate, clozapine and risperidone administrations significantly (p<0.01) decreased HVA/DA ratio and increased DOPAC/DA ratio in nucleus accumbens at all doses. The findings suggest the evidence for DA/5-HT receptor interaction as an important link in the lower incidence of EPS. The possible role of serotonin_1A_ receptors in the pathophysiology of schizophrenia is also discussed.

## Introduction

Schizophrenia is a clinical syndrome with diverse manifestations. Dopamine (DA) D_2_ receptor antagonism is postulated to be a key to antipsychotic efficacy in the treatment of schizophrenia [1]. Typical antipsychotic drugs with a high extrapyramidal motor side-effects (EPS) liability markedly increase extracellular dopamine in the caudate-putamen, while atypical antipsychotic drugs with a low incidence of EPS have less pronounced stimulating actions on corpus striatal dopamine [2, 3]. Therefore, it has been suggested that the EPS liability of antipsychotic drugs (APD) is correlated with their ability to increase extracellular dopamine in the caudate-putamen [3]. The caudate nucleus is highly innervated by dopamine neurons. These neurons originate mainly from the ventral tegmental area (VTA) and the substantia nigra pars compacta (SNc) [4]. There are also additional inputs from various association cortices. Nucleus accumbens (NAcc) is another basal ganglia structure probably mediating EPS of typical antipsychotic drugs [5]. Therefore, the present study sought to determine whether extracellular dopamine in the NAcc might be a further indicator to differentiate neurochemical actions of typical and atypical antipsychotic drugs. The ability of antipsychotic drugs to affect 5-HT_1A_ receptor function has been widely suggested to contribute to their therapeutic properties [6]. Atypical antipsychotic drugs related to clozapine, improve psychosis, cognition and negative symptoms, while producing minimal EPS, in patients with schizophrenia [7]. This appears to be mediated mainly through the combined effect of relatively more potent blockade of 5-HT_1A_ receptors, located on cortical and hippocampal glutamatergic and GABAergic neurons, as well as cell bodies of the mesolimbic and mesocortical dopamine (DA) neurons, and weaker blockade of D_2_ receptors in the ventral and dorsal striatum and pyramidal neurons in cortical areas, as well as the cell bodies of DA neurons [7, 8]. The implications of EPS reduction touch virtually every domain of pathology in schizophrenia, including short- and long-term movement disorders, negative symptoms, noncompliance, relapse rate, cognitive dysfunction, and dysphoria [9]. The important goal of the present research on atypical neuroleptics is to develop novel compound(s) with antipsychotic potential with a low incidence of EPS.

A majority of schizophrenic patients respond to classical neuroleptic drugs which are believed to act largely via blockade of dopamine (DA) D_2_ receptors in limbic structures [10]. However, a significant proportion do not respond to conventional D_2_ receptor blocking drugs or show a rather incomplete therapeutic effect, particularly on negative and cognitive symptoms [11]. An examination of clinical literature shows that haloperidol is very likely to produce EPS, clozapine is relatively unlikely and risperidone is somewhat mild in terms of the production these effects [12]. In comparison with classical neuroleptics atypical antipsychotic drugs such as for example clozapine and risperidone have been found to posses improved efficacy against negative symptomatology and cause a reduced incidence of EPS in clinically effective doses [13]. The present work was undertaken in an attempt to gain further insights into the mechanisms of action of atypical antipsychotic drugs as an icon with low propensity towards EPS in rats. Clozapine, the first of the atypical antipsychotic, has been reported to be effective in approximately one third or two third of patients with treatment resistant disease [12, 13]. The atypical is reported to be effective for the treatment of both positive and negative symptoms of schizophrenia. It also produces smaller EPS than typical neuroleptics. However, clozapine has the potential for fatal adverse effect: agranulocytosis [14], a sudden drop in white cell count that could leave the patient vulnerable to overwhelming infections [15]. Obviously, a goal of current neuroleptic research is to develop antipsychotic compounds with low incidence of EPS and agranulocytosis. One of the approaches in this context has been to determine neurochemical/ pharmacological correlates of clozapine and other atypical neuroleptics. A distinguished element of the pharmacology of clozapine is its affinity for serotonin (5-hydroxytryptamine; 5-HT) receptors particularly 5-HT_2A_, 5-HT_2C_ and 5-HT_1A_ receptors [16, 17]. The atypical has lower affinity for dopamine (DA) D_2_ receptors than traditional antipsychotics [4, 11]. Although clozapine is recognized as the prototypal atypical anti-psychotic drug, there are no well defined criteria for classifying newly developed antipsychotics as atypical. Meltzer [12] suggested defining an antipsychotic as atypical based on antagonistic actions at both, D_2_ and 5HT_2_ receptors as well as clinical criteria of minimal induction of EPS at therapeutic doses.

The present contribution focuses on the possible role of different 5-HT receptors in the modulation of atypical neuroleptic mechanism of action other than dopamine receptors. Following the discovery of 5HT_2_ (now termed 5HT_2A_) receptors, it was reported that several neuroleptics bound to both 5HT_2A_ and D_2_ receptors [12, 13]. Risperidone provided further evidence that potent 5HT_2A_ antagonism combined with milder D_2_ antagonism [12, 13] resulted in significantly improved clinical properties [12, 13]. Indeed, in addition to successful treatment of the positive symptoms, risperidone markedly improved the negative symptoms of schizophrenia with a low liability of EPS [14–16]. Risperidone, a benzisoxazol derivative, is an antipsychotic agent, which combines potent serotonin 5HT_2_ and dopamine D_2_ receptor antagonism [14, 15] and has been introduced in the wake of clozapine induced toxicity. Moreover, clinical studies have revealed that risperidone displays a beneficial antipsychotic action, improving both positive and negative symptoms, while having a low propensity to induce EPS [16, 17]. Besides 5-HT_2_ and DA-D_2_ receptors, risperidone exhibits rather high affinity for central α_1_ adrenergic and H_1_ histamine receptors and a moderate affinity for α_2_ adrenergic receptors [12, 14–16]. Tissue measurements of regional DA and 5-HT metabolism in the rat brain have indicated that risperidone affects DA turnover in both mesolimbic and nigrostriatal terminal regions and, to some extent, also 5-HT metabolism in these target areas [12].

The aim of the present study was, therefore, to compare the dose-response effects of clozapine and risperidone on extrapyramidal motor functions and on DA metabolism in rat caudate and nucleus accumbens. Homovanillic acid (HVA) and dihydroxyphenyl acetic acid (DOPAC) are the predominant metabolites of DA, Therefore, in the present study effects of clozapine and risperidone are also compared on HVA/DA and DOPAC/DA ratios in caudate and nucleus accumbens of a rat brain.

## Materials and Methods

### Experimental Animals

The protocol for experimentation was approved and performed in strict accordance with the Guide for the Care and Use of Laboratory Animals (Institute of Laboratory Animal Resources on Life Sciences, US National Research Council, 1996) and the Institutional Animal Ethics Committee (IAEC). Male Albino-Wistar rats with an average weight of 180±20 g, purchased from Agha Khan University (AKU) were fed on standard diet and water *ad libitum*. The animals were group-housed (two rats per cage) in an environmentally controlled room (ambient temperature 24±2°C and relative humidity 55±5%) on a 12:12 h light/ dark cycle (lights on at 7:00 A.M.). A 5-day acclimatization period was allowed before animals were used in experiments. After this period, and 24 h before the behavioral tests, the animals were individually housed in an environmentally controlled test room in transparent Perspex cages (dimensions 26×26×26 cm W×L×H). The guidelines of IAEC, Pakistan the governing body for animal experimentations in Pakistan, were strictly adhered to during the whole animal experimentation protocol.

### Drugs and Injections

Clozapine (Sigma) was dissolved in a minimum volume of 0.1 M acetic acid and pH adjusted to 6.5 by NaOH and diluted with physiological saline (0.9% NaCl) to concentrations of 2.5 mg/ml, 5.0 mg/ml and 10.0 mg/ml IP in volumes of 1ml/kg body weight. Risperidone (Janssen) was dissolved in 0.4M tartaric acid and pH adjusted to 6.5 by NaOH and diluted with saline to the desired concentrations of 1.0 mg/ml, 2.5 mg/ml and 5.0 mg/ml IP in volumes of 1.0 ml/kg body weight. Control animals received equal volume of vehicle (0.1 M acetic acid, 0.1 M NaOH and/or 0.4 M tartaric acid and saline in the ratio of 1: 1: 8). The animals were injected in a balance design between 10:00 and 11:00 h. Motor activity monitored for 10 min starting 10 min after injections. Catalepsy effects of the drugs were scored for a cut off time of 3 min starting 30 min post injections and animals were killed to collect brain samples 1 h after injections.

### Behavioral Procedures

#### Dose-Dependent Effects of Clozapine and Risperidone on Locomotion

Motor-related effects of the drugs were monitored in Perspex activity cages. Animals were transferred in Perspex activity cages (26x26x26 cm) with saw dust covered floor 15 min before injecting the saline or drug. Experiment was conducted in a separate quiet room. Motor activity in the activity box was monitored under white light as numbers of cage crossings/ 10 min starting 10-min post injections in all groups of rats in a balanced design [18].

#### Dose-Dependent Effects of Clozapine and Risperidone on Catalepsy

Catalepsy, defined as the acceptance and retention of abnormal posture, was measured by means of a bar test. Bar test determinations were carried out by gently removing rats (n=12) from their home cages and placing their forepaws over a horizontal bar, fixed at a height of 10 cm with heads of animals upwards on an inclined surface at an angle of 60° with the hind limbs abducted [4, 18]. The lapse of time during which the animal retained this position was recorded by measuring the time from the placement of the rat until removal of one of its forepaws. An animal to be tested was placed on an inclined surface (26 x 40cm, 60° inclination) for cut off time 3 min. A control rat changes its position immediately on the inclination. Time (sec) as drug or saline injected animals remained immobile on the inclination was monitored. Animals not moving on the surface up to 3 min were kept back in their cages. Cataleptic score was calculated as {Latency of movement (s)/ cut-off time (180 s)} 100.

### Neurochemical Analysis

#### Dissection of Caudate and Nucleus Accumbens

Saline, clozapine or risperidone injected animals were killed 1 h post injections. The dissection procedure was essentially same as described before [18, 19]. A fresh brain was dipped in ice-cold saline and placed with its ventral side up in the molded cavity of a brain slicer. Fine fishing line wire was inserted into the slots of the slicer to give slices of 2 mm thickness. The slice containing caudate and nucleus accumbens was transferred to a glass plate kept on ice. Punches of 2.5 mm diameter were made bilaterally in the upper and lower sides of the slice to collect the desired brain regions. The striatum also known as striate body, neostriatum or striate nucleus is a subcortical (i.e., inside, rather than on the outside) part of the telencephalon/cerebrum. It is the major input station of the basal ganglia system. In primates (including humans), the striatum is divided by a white matter tract called the internal capsule into two sectors called the *caudate nucleus (corpus striatum) and nucleus accumbens (ventral striatum)*. The collected brain regions were immediately stored for the biogenic amine assay at −70°C for the estimation of DOPAC, DA, HVA and 5-HIAA levels by High performance liquid chromatography with electrochemical detection (HPLC-EC) [18].

### HPLC-EC Analysis

Biogenic amines and their major metabolites were extracted with 150 μl of perchloric acid (HClO_4_; 70 %) from brain tissue punches (< 250 μg) using a simple one-step sample preparation method (19). A 5 μm (particle size) ODS column (4.0 mm i.d. and 250 mm length) was used. Mobile phase comprised of proportions of methanol (CH_3_OH; 14 %), octyl sodium sulphate (OSS; 0.023 %) and ethylenediaminetetraacetic acid (EDTA; 0.0035 %) in 0.1M phosphate buffer of pH 2.9 was passed through the column at constant flow rate (1.0 ml/min) with the help of Waters 510 HPLC pump (Waters Corporation, US). Electrochemical detection was achieved on Schimadzu L-EC 6A detector (Shimadzu Corporation, Japan) at an operating potential of 0.8 V. Tryptophan was determined in a separate run at an operating potential of 1.0 V (18, 19).

### Statistical Analysis

Results are presented as means ± S.D. Statistical analysis was performed using one-way ANOVA (analysis of variance) and *Post hoc* comparison was done by the Newman-Keuls test. *p* values < 0.05 were considered statistically significant.

## Results

**Fig. 1.**
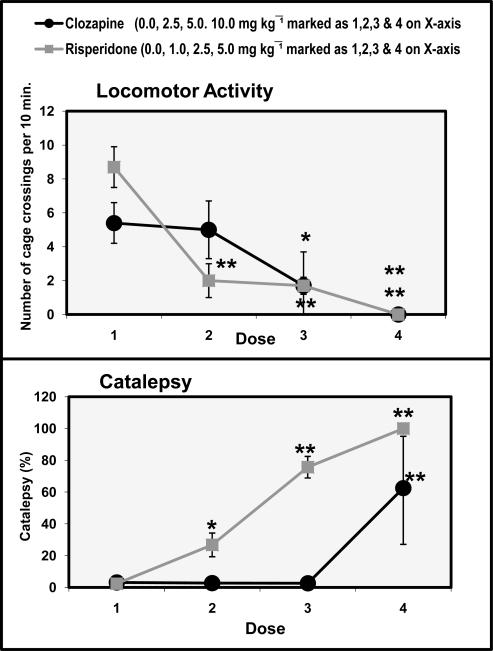
Dose-response effects of clozapine and risperidone on locomotor activity and cataleptic potential in rats. Values are mean ± SD (n=12). Significant differences by Newman-Keuls test *p<0.05, **p<0.01 as compared to respective saline-treated rats following ANOVA.

Figure 1 shows the dose-response relationship of clozapine and risperidone on locomotor activity and cataleptic potential in rats. ANOVA (df 3,12) showed significant effects of risperidone (1.0, 2.5 and 5.0 mg/kg) on motor activity (F=65.7 p<0.01) and catalepsy (F=226.9 p<0.01). Effects of clozapine (2.5, 5.0 and 10.0 mg/kg) on motor activity (F=8.0 p<0.01) and catalepsy (F=9.8 p<0.01) were also significant. *Post hoc* analysis showed that 100% catalepsy occurred only at a dose of 5.0 mg/kg of risperidone and 60% at 10.0 mg/kg of clozapine. Clozapine did not decrease activity at a dose of 2.5 mg/kg but decreased it at doses of 5.0 mg/kg and 10.0 mg/kg. No catalepsy was observed at other doses of clozapine and risperidone.

**Fig. 2.**
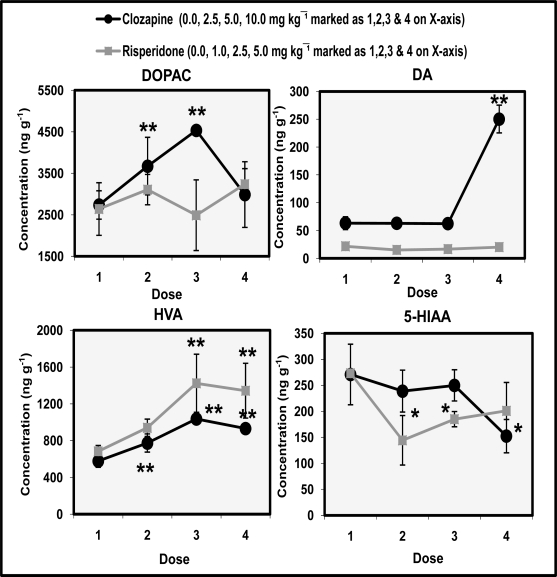
Dose-response effects of clozapine and risperidone on DOPAC, DA, HVA and 5-HIAA in rat caudate. Values are mean ± SD (n=12). Significant differences by Newman-Keuls test *p<0.05, **p<0.01 as compared to respective saline-treated rats following ANOVA.

Figure 2 shows the dose-response effects of clozapine and risperidone on the levels of dopamine (DA), metabolites dihydroxyphenylacetic acid (DOPAC) and homovanillic acid (HVA), and 5-hydroxyindoleacetic acid (5-HIAA) in the caudate nucleus. ANOVA (df 3,12) showed significant effects of clozapine on the levels of DA (F=129.7 p<0.01), HVA (F=30.7 p<0.01), DOPAC (F=6.3 p<0.05) and 5-HIAA (F=4.6 p<0.05). Effects of risperidone were also significant for HVA (F=7.1 p<0.05). Effects of risperidone on DA (F=1.2 p>0.05), DOPAC (F=1.1 p>0.05) and 5-HIAA (F=1.2 p>0.05) were not significant. *Post hoc* analysis showed effects of risperidone on DOPAC changes were not significant. Rats injected with 2.5 mg/kg and 5.0 mg/kg exhibited pronounced 2–3 fold increases in HVA concentration and these increases were also comparable. HVA levels were also increased following the administration of clozapine and the increases maximum at a dose of 5.0 mg/kg. Maximum increases of HVA were greater in risperidone than clozapine injected animals. The concentration of DOPAC also increased following the administration of clozapine at a dose of 2.5 mg/kg and 5.0 mg/kg. DOPAC levels did not increase at cataleptogenic dose (10.0 mg/kg) of clozapine. 5-HIAA levels increased following the administration of high dose of risperidone but these increases were not significant. Conversely, clozapine administration did decreased 5-HIAA concentration only at high dose of 10.0 mg/kg. The decreases not significant at doses of 2.5 mg/kg and 5.0 mg/kg were significant (p<0.01) at the cataleptogenic dose of 10.0 mg/kg.

**Fig. 3.**
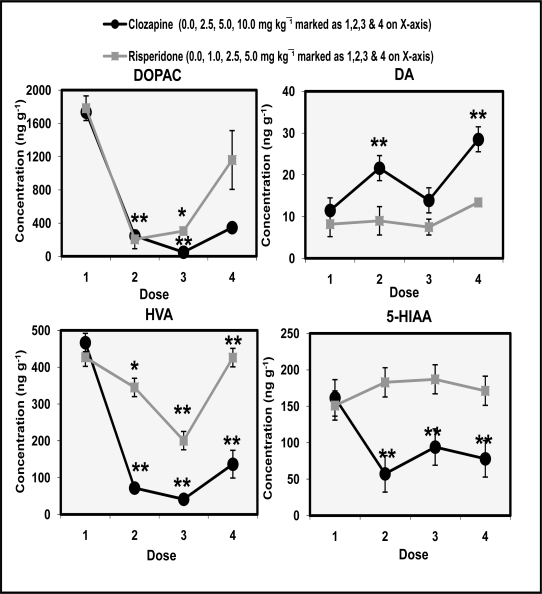
Dose-response effects of clozapine and risperidone on DOPAC, DA, HVA and 5-HIAA in rat nucleus accumbens. Values are mean ± SD (n=12). Significant differences by Newman-Keuls test *p<0.05, **p<0.01 as compared to respective saline-treated rats following ANOVA.

Figure 3 shows the dose-response effects of clozapine and risperidone on the levels of DOPAC, DA, HVA and 5-HIAA in rat brain nucleus accumbens. ANOVA (df 3,12) showed significant effect of clozapine on DOPAC (F=64.5 p<0.01), DA (F=15.6 p<0.01), HVA (F=19.5 p<0.01) and 5-HIAA (F=14.5 p<0.01) concentrations. Effects of risperidone were significant for DOPAC (F=12.5 p<0.01) and HVA (F=27.8 p<0.01). Non-significant effects of risperidone were observed for DA (F=1.7 p>0.05) and 5-HIAA (F=34.6 p<0.01). *Post hoc* comparison showed that clozapine significantly (p<0.01) decreased concentrations of DOPAC, HVA and 5-HIAA in a dose-dependent manner. Levels of DA significantly (p<0.01) significantly increased at doses of 2.5 mg/kg and 10.0 mg/kg of clozapine. Levels of DA and 5-HIAA remained insignificant at all the doses of risperidone.

**Fig. 4.**
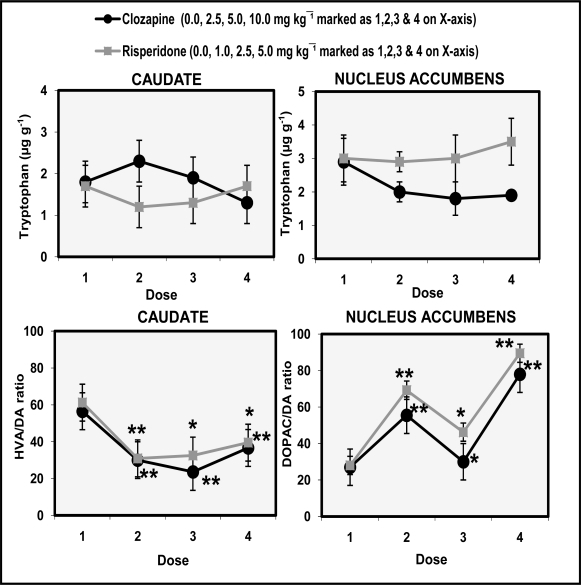
Dose-response effects of clozapine and risperidone on Tryptophan, HVA/DA and DOPAC/DA ratios in rat caudate and nucleus accumbens. Values are mean ± SD (n=12). Significant differences by Newman-Keuls test *p<0.05, **p<0.01 as compared to respective saline-treated rats following ANOVA.

Figure 4 shows the dose-response effects of clozapine and risperidone on the levels of tryptophan and the ratios of HVA/DA and DOPAC/DA in the rat caudate and nucleus accumbens. ANOVA (df 3,12) performed on caudate data showed insignificant effect of clozapine (F=2.1 p>0,05) and risperidone (F=4.6 p<0.05) for TRP. ANOVA (df 3,12) performed on data of nucleus accumbens also showed insignificant effect of clozapine (F=0.49 p>0,05) and risperidone (F=3.37 p>0.05) for TRP. Data analyzed for caudate showed significant effects of clozapine for HVA/DA (F=15.1 p<0.01) and risperidone (F=33.2 p<0.01). Data on nucleus accumbens showed significant effect of clozapine on DOPAC/DA ratio (F=10.34 p<0.01) and risperidone (F=9.46 p<0.01). *Post hoc* analysis showed that the caudate ratio of HVA/DA decreased significantly (p<0.01) in a dose-dependent manner. Conversely, levels of DOPAC/DA ratio were significantly (p<0.01) increased in nucleus accumbens by all the doses of clozapine and risperidone.

## Discussion

In the present study, atypical antipsychotic drugs, or so-called second-generation antipsychotics, like clozapine and risperidone, are defined as prototype agents causing low incidence of EPS in laboratory animals at doses that demonstrate antipsychotic activity. Management of extrapyramidal side effects (EPS) is a major concern of clinicians who use antipsychotic drugs. The incidence of motor side effects is dose dependent. The distinction between typical and atypical antipsychotic drugs has attained a considerable importance now. Moreover, the 5-HT_2A_/D_2_ hypothesis has contributed to the development of most of the current generation of atypical antipsychotic agents, including risperidone, olanzapine, quetiapine, ziprasidone, melperone and perospirone, all of which have a higher affinity for 5-HT_2A_ than for D_2_ receptors [16, 27–31]. Atypicality has been ascribed to 5-HT_2_/ D_2_ antagonism that may be involved with the antipsychotic effect [2, 20]. The typical effect of administering a DA-D_2_ receptor antagonist is a suppression of spontaneous locomotor behavior and elicitation of a state known as catalepsy in laboratory animals [4, 22]. In the present study, all the doses of clozapine and risperidone we used were found to decrease motor activity in a dose-dependent manner (Fig. 1). Both drugs were found cataleptogenic only at high doses. However, effects of both drugs on locomotor activity in a familiar environment were comparable at all the doses.

A major finding of the present study is that acute administration of risperidone relatively uniformly increases DA release and metabolism in the caudate region of rat brain. It is now generally agreed that clinical response to neuroleptics is associated with an increase in HVA concentration [24]. The proposed mechanism of HVA changes involves acute blockade of pre and postsynaptic D_2_ receptors in caudate. The presynaptic blockade increases the amount of DA released on subsequent action potentials, but because D_2_ postsynaptic receptors are also blocked, acute DA transmission is attenuated. In addition, it is possible that the relative lack of EPS motor effects with clozapine and risperidone administration in the present study could be related to two-way interaction of drugs towards DA and 5-HT receptors.

It is suggested from the present findings that the agents increasing dopamine transmission inhibits neuroleptic-induced catalepsy. The caudate and nucleus accumbens have been implicated as the major brain structures involved in antipsychotic induced catalepsy, which appears due to the blockade of dopamine neurotransmission. Animal studies suggest that the nucleus accumbens is the critical region in which both typical and atypical antipsychotic drugs exert their antipsychotic effects [25]. It was shown in early studies that administration of antipsychotic drugs increased DA turnover in rat brain [25]. Later studies, generally addressed DA release, show an increase in DA release in regions rich in DA terminals [23, 25]. It has been shown that blockade of pre- and postsynaptic receptors by a single injection of neuroleptics leads to an increase in the spike discharge of neurons belonging to the nigrostriatal system of the rat brain. It has been suggested that increased DA release in the caudate under the influence of neuroleptics can make a definite contribution to the formation of atypical pharmacologic profiles of these substances due to competition between the released mediator and neuroleptics at the level of postsynaptic DA receptors [23].

The present study shows that administration of both clozapine and risperidone increased HVA concentration in the caudate (Fig. 2). Risperidone was more effective than clozapine in producing this effect in the caudate region. DOPAC the other metabolite of DA increased at noncataleptogenic doses of clozapine whereas, risperidone did not alter DOPAC concentration in the caudate. 5-HIAA levels were decreased at cataleptogenic dose (10.0 mg/kg) of clozapine and noncataleptogenic doses (1.0 & 2.5 mg/kg) of risperidone in caudate. Conversely, administration of clozapine and risperidone significantly decreased levels of DOPAC and HAV in the nucleus accumbens dose-dependently (Fig. 3). 5-HIAA levels were also significantly decreased by clozapine administration in a dose-dependent manner. Cataleptogenic dose (10.0 mg/kg) of clozapine significantly increased DA levels in nucleus accumbens. Overall impact of clozapine and risperidone administration suggests that DA metabolism in caudate region was more responsive in evaluating the cataleptic potential of the drugs as compared to nucleus accumbens. Both drugs did not alter levels of TRP in caudate and nucleus accumbens. However, significant dose-dependent decreases were observed in caudate HVA/DA ratio following the administration of clozapine and risperidone. On the other hand, DOPAC/DA ratio in the nucleus accumbens were significantly increased at all the doses of clozapine and risperidone (Fig. 4). The present results also show that clozapine-induced increases of DA metabolism largely take place in the caudate than in the nucleus accumbens. Comparable effects of clozapine and risperidone on DA metabolism are at least, partly explicable in terms of their activities at DA and 5-HT receptors. Clozapine has a lower affinity for D_2_ receptors and risperidone has high affinity for 5-HT_2_ receptors. In vitro data indicate that clozapine is approximately equipotent at D_1_ and D_2_ receptors [27]. Other authors have also reported regional differences in D_2_ affinity of clozapine [28]. A role of D_4_ receptors has also been implicated in the mechanism of action of clozapine. The drug has high affinity for D_4_ receptors, higher than its affinity for D_2_ receptors [27, 28]. Another distinguished feature of the pharmacology of clozapine is its high affinity for serotonin receptors particularly 5-HT_2A_ and 5-HT_2C_ sites. This pharmacological profile of the drug is often linked with its therapeutic profile and lower incidence of EPS as observed in present study.

There is now considerable evidence from studies of schizophrenia and other forms of psychoses, e.g., senile psychoses and L-DOPA psychoses, that a number of atypical antipsychotic agents such as clozapine and risperidone have also been found to have greater affinity for 5-HT_2A_ than D_2_ receptors *in vitro* and are more effective with fewer side effects. The serotonergic effects of clozapine also have been emphasized as possibly being related to the unique clinical characteristics of clozapine [31], and recently it was suggested that the 5-HT_2A_/ D_2_ binding ratio could be used to distinguish between typical and atypical antipsychotics [32]. A decrease in 5-HT turnover by clozapine is explicable in terms of drugs moderate affinity for 5-HT_1A_ receptors [32, 33]. Antipsychotic-like effects of clozapine are also often explained in terms of 5-HT_1A_ agonist activity of the drug [33]. 5-HT_1A_ agonists decrease 5-HT turnover in various brain regions because of the stimulation of receptors located on cell body dendrites [34]. There is evidence that 5-HT_1A_ agonist activity is involved in clozapine’s beneficial clinical profile and lower incidence of EPS. Thus postsynaptic 5-HT_1A_ receptors were up regulated in postmortem schizophrenic patients [34]. 5-HT_1A_ agonists have been shown to reverse haloperidol-induced catalepsy [35]. A comparison of the effects of two drugs shows that an increase in HVA concentration in the caudate (Fig. 2) and an increase in DOPAC/DA ratio in nucleus accumbens (Fig. 4) may be taken as a neurochemical profile of atypical, clozapine like, activity. Other authors have reported significant increases in caudate-putamen volumes in animals receiving either haloperidol or clozapine when compared with control animals following eight months of drug administration [36]. It is suggested that the neuroplastic response of the striatum following neuroleptic exposure causes volumetric increases, whereas atypical antipsychotics affect the basal ganglia differentially suggesting that such differential responses may be due to both the pharmacological properties and the relative doses of the atypical agents.

In conclusion, the present study shows that administration of both clozapine and risperidone increased DA metabolism more in caudate than in nucleus accumbens in a dose-dependent manner. Consequently, our findings demonstrate a pharmacological profile of risperidone, as reflected in brain DA metabolism, with that of clozapine as a reference atypical compound. The preferential activation of DA release and metabolism in caudate and nucleus accumbens areas might be of particular relevance for the ameliorating effect of risperidone on negative symptoms in schizophrenia, especially when associated with depression. It is imperative to monitor for the emergence of EPS and TD in future studies, so that patients are not distressed or uncomfortable, and to facilitate adherence with ongoing antipsychotic therapy in patients with schizophrenia or other major psychiatric illnesses.
